# The Use of Artificial Intelligence in Dentistry Practices

**DOI:** 10.5152/eurasianjmed.2022.22301

**Published:** 2022-12-01

**Authors:** Ozkan Miloglu, Mustafa Taha Guller, Zeynep Turanli Tosun

**Affiliations:** 1 Department of Oral, Dental and Maxillofacial Radiology, Atatürk University Faculty of Dentistry, Erzurum, Turkey; 2 Department of Dentistry Services, Oral and Dental Health Program, Binali Yıldırım University Vocational School of Health Services, , Erzincan, Turkey

## Abstract

Artificial intelligence can be defined as “understanding human thinking and trying to develop computer processes that will produce a similar structure.” Thus, it is an attempt by a programmed computer to think. According to a broader definition, artificial intelligence is a computer equipped with human intelligence-specific capacities such as acquiring information, perceiving, seeing, thinking, and making decisions.

Quality demands in dental treatments have constantly been increasing in recent years. In parallel with this, using image-based methods and multimedia-supported explanation systems on the computer is becoming widespread to evaluate the available information. The use of artificial intelligence in dentistry will greatly contribute to the reduction of treatment times and the effort spent by the dentist, reduce the need for a specialist dentist, and give a new perspective to how dentistry is practiced. In this review, we aim to review the studies conducted with artificial intelligence in dentistry and to inform our dentists about the existence of this new technology.

Keywords: Deep learning, dentistry, machine learning, neural networks, artificial intelligence

## Introduction

Artificial intelligence (AI) is a term that describes a computer that imitates human intelligence. Human intelligence may sometimes be flawed, but it can cope with many complex situations. Artificial intelligence is a method that aims to model the way the human brain works and can be imitated through computers.^1^

Artificial intelligence is now used in audio, video, autonomous systems, social networks, shopping, construction, computer and robot engineering, economics, factories, agriculture and livestock, energy, medicine, and many other fields.^1,2^ Thus, the need for human power and intelligence is reduced, and human-related errors are minimized. Studies in the medical field are generally on the prediction of the diagnosis and/or prognosis of diseases. For example, in the diagnosis of coronavirus disease-2019, a rapid decision can be made for diagnosis from lung x-ray or lung computed tomography (CT) images using AI.^1,2^

Correct diagnosis in dentistry can be made with the help of many methods, such as extraoral or intraoral radiographs, advanced imaging systems, computer technologies, and advanced examination techniques. Despite all these tools, the actual decision is made by the physician.^1,2^

Correct diagnosis is very important for a successful treatment. Using AI technologies in the health field can reduce human-related errors, reduce the time for diagnosis, and reduce the need for experts. In this sense, there are many AI techniques, and the power of these techniques to diagnose or solve problems is still being studied. In this review, we aim to reveal the potential of AI in dentistry education and clinical practice, to facilitate faster adoption of AI techniques, and to provide a reference to future studies.

## Restorative Dentistry and Artificial Intelligence

Tooth decay is one of the most common oral diseases. Early diagnosis of tooth decay is important for the long-term preservation of natural teeth. In AI models performed in this area, tooth decay was detected on intraoral photographs and dental radiographs.^3,4^ In their study, Moutselos et al^5^ reported that they detected occlusal tooth decay in the images obtained with the intraoral camera using the region-based convolutional neural network (CNN) model. Lee et al^6^ evaluated the detection of tooth decay in periapical radiographies with the CNN model in their study. They found the accuracy rate to be 89.2% for premolar teeth, 88.5% for molar teeth, and 82.3% for both premolar and molar teeth. In other studies, models developed with radiographic data that can detect tooth decay in extracted teeth are presented.^7^ Thus, early diagnosis of tooth decay helps to keep the teeth in the mouth for a long time and reduces the cost of dental treatments.

Restorative dental treatment practice includes permanent and temporary filling materials, adhesive systems, prophylactic applications, high-speed rotating tools, and hand tools used in cavity preparation.^8^ In their study, Zakeri et al^9^ analyzed the sounds of high-speed rotating tools used in dentistry when in contact with teeth and restoration materials and studied the distinction of these sounds. In the study, amalgam and composite were used as restoration materials, and it was aimed to help dentists by preventing accidental loss of substance from dental tissue during the removal of restorations. Aliaga et al^10^ tried to determine the most suitable restoration material (amalgam or composite) for the cavity with the AI model they developed using their analysis and radiological information on restorative treatments performed in the past years. In another study in which a model was developed for the detection and classification of dental restorations in panoramic radiographs (PR), 11 types of restorations over 83 PR were determined, and the rate of detecting the restoration of the model was found to be 94.6%.^11^ In another study, backpropagation and genetic algorithm methods were combined, and a method that can provide more accurate estimates in matching the materials used in dental restorations with natural tooth color was developed.^12^

### Endodontics and Artificial Intelligence

Adequate chemo-mechanical preparation and effective filling of the root canal system in endodontics practice are closely related to the detailed knowledge of root canal morphology. Failure to treat all channels effectively leads to poor endodontic outcomes and reduces treatment success. In this sense, conical beam computed tomography (CBCT) has recently been used to evaluate root canal morphology. Conical beam computed tomography is an imaging method that offers noninvasive and 3-dimensional reconstruction in endodontic applications and morphological analyses by clinicians. However, the use of CBCT poses a high risk due to the excessive exposure of patients to ionizing radiation.^13^ As a newer method, AI studies have begun to be conducted on subjects such as the detection and location of canal orifices, the location of anatomical and radiological apical foramen, and the determination of the anatomical shape of the root canal in endodontics applications.^2^

Endodontic treatment aims to eliminate microorganisms and residues and to prepare the root canal system for obturation. The narrowest part of the canal is called apical stenosis, and ideally, the apical end of the preparation should be at this point. Radiographs are used to determine the end point of clinical procedures. Misinterpretation of radiographs leads to incorrect determination of working length. Determination of the working length in endodontics studies is one of the most important steps. Failure to determine the working length may cause insufficient or excessive root canal instrumentation.^14^ Since the location of the radiological apical foramen may differ among clinicians, a second opinion can be obtained with the help of AI to contribute to the increase in the success of canal treatment. Studies have reported that artificial neural networks (ANNs) can be used to determine the radiographic location of the apical foramen and can help determine the working length in canal treatment.^2^

The software has been developed using augmented reality to perform real-time canal orifices detection and teeth classification through video images.^15^ With this software, it has been reported that the number and location of the canal orifices of the detected teeth can be stored as data, which can help future statistical studies. Yang et al^16^ evaluated the quality of canal treatment from periapical radiographs with the CNN model. Using micro-CT and CBCT image data, algorithms that can define the 3-dimensional (3D) image of the root canal system and automatically detect the root canals and medial line have been developed.^17^

The AI model was performed to classify the root morphology of mandibular first molar teeth in PRs. In this study, an extra root in the distal root of the first molar teeth was labeled in PRs by examining CBCT images. It has been reported that AI models are 86.9% accurate in detecting the presence of extra roots in the distal root in PRs.^18^ The C-shaped canals are variations generally seen in mandibular second molar teeth, difficult to detect in 2-dimensional radiographs, and reduce the success of endodontic treatment. In a study, 95.1% accuracy was obtained in the CNN model formed by scanning CBCT images to predict C-shaped channels in PRs.^19^

An important cause of endodontic failure is root fracture. This is a serious clinical problem and may result in tooth extraction or resectioning of the affected root.^20^ Many complications, such as zipping, stepping, or moving root tips, may occur, especially during the shaping or refilling of the crooked teeth. These complications may cause the root to weaken and apical blockage to fail.^21^

Vertical root fractures are more difficult to detect with 2-dimensional radiographs than with 3D imaging systems.^22^ Kositbowornchai et al^23^ used the ANN model in intraoral radiographs to detect vertical root fractures. The presence of only premolar and single-rooted teeth in the data set is an obstacle to the use of the study in routine clinical practices. Further studies will contribute more to clinical applications by including different teeth groups. In a similar study, ANN was used to detect vertical root fractures in periapical and CBCT images of premolar teeth, and it was found that AI performance was better in CBCT images.^24^ Fukuda et al^25^ developed a CNN model using 300 PR that can detect vertical root fractures in different teeth.

## Oral Pathology and Artificial Intelligence

Due to the high number of cysts and tumors in the maxillofacial region and their similar radiological appearance, it may be difficult to make a differential diagnosis of these lesions.^26^ Advanced imaging methods such as radiographs or CBCT and ultrasonography are frequently used to diagnose lesions in this region. It is widely preferred for imaging of maxillofacial regions since CBCT has advantages such as showing hard tissues well and less radiation than medical CT.^27^ Ultrasonography is generally used in dentistry to evaluate salivary gland diseases, foreign bodies in the soft tissues in the orofacial region, orofacial muscles, tongue lesions, and lymph nodes.^28^ In order to make a more accurate radiological preliminary diagnosis, various studies have been conducted using systems developed with AI. In light of these studies, AI can give clues that can help clinicians in radiological diagnosis. Segmental analysis of cysts and tumors helps determine the location and size of the relevant structure.^29^ Abdolali et al^30^ developed an AI model that segmented and based this distinction on asymmetry analysis in a study in which CBCT images of the radicular cyst, dentigerous cyst, and odontogenic keratocysts were included in the data set. In their study, Rana et al^31^ compared manual, threshold-based, and automatic segmentation methods in terms of time and accuracy by using the image data of cases diagnosed with odontogenic keratocyst. They reported that automatically performing segmentation (smart brush) provided reliable and fast results. In another study, an algorithm that can perform epithelial segmentation in digital micrographs of hematoxylin eosin-stained samples of 4 odontogenic cysts (dentigerous cyst, lateral periodontal cyst, odontogenic keratocyst, and glandular odontogenic cyst) was developed.^32^ In addition to the previous research, the same team developed a fully automated algorithm that can define the difference of epithelial layers of four different odontogenic cysts by using a support vector machine (SVM) and logistic regression and thus classify cysts.^33^ In another study, the correct diagnosis of 3 types of odontogenic cysts (odontogenic keratocyst, dentigerous cyst, and periapical cyst) in PR and CBCT images was investigated with the CNN model developed. The accuracy of the model obtained from CBCT images was 91.4%, and the accuracy obtained from PR images was 84.6%.^34^ Although it is difficult to diagnose accurately with radiological data, this rate can be increased with AI. Ameloblastoma and odontogenic keratocyst are common lesions in the jaws. The radiological features of these lesions are similar, and preoperative detection helps in treatment planning. A CNN model has been developed that distinguishes these 2 lesions in PRs.^35^ In another study, a deep learning (DL) method was developed that can distinguish into 4 categories: dentigerous cyst, ameloblastoma, odontogenic keratocyst, and non-lesional.^36^

A CNN model has been developed to distinguish normal tissues from pathological tissues using auto-fluorescent and white light images obtained by intraoral scanning devices of malignancies and dysplasias in the oral region.^37^ By expanding the dataset of this study and adding more pathologies, automatic detection of pathologies in the oral region using AI can help clinicians more. In this context, in a study developed with hyperspectral images, in which the DL algorithm was used, for the early detection of oral cancers, an accuracy of 91.4% was determined.^38^ In another study, intraoral squamous cell carcinoma (SCC) detection was made using photographic images.^39^ In addition, a model has been developed that can detect oral SCC with an accuracy of 99.4% by looking at the shape, texture, and color features in histopathological images.^40^ In their study, Wu et al^41^ developed an algorithm that detects SCC diagnosis with high accuracy in positron emission tomography/CT images that detect lesions larger than 1 cm and have a high metabolism. In addition to diagnosing SCC, various machine learning (ML) techniques are used to predict the survival of individuals with related pathologies.^42,43^

Bisphosphonate group drugs used in some bone-related diseases may cause osteonecrosis (bisphosphonate-related osteonecrosis, BRONJ) in the jaws after tooth extraction. In the study, 5 different ML methods were used that can predict the probability of BRONJ formation in the jaws after tooth extraction in patients using bisphosphonates for the treatment of osteoporosis, and it was found that ML performances were superior to statistical data such as serum carboxyterminal peptide level and drug discontinuation.^44^ They also stated that the best performance in ML models is the random forest (RF) algorithm.

### Periodontology and Artificial Intelligence

Periodontal diseases are an important public health problem due to their high prevalence and may lead to the loss of teeth. Scaling and root planning form the basis of periodontal treatment. These procedures are usually performed with hand tools and aim to remove not only the debris on the root surface but also the dental tissue, albeit in small amounts.^45^

Artificial intelligence models have been developed in the radiological diagnosis of diseases occurring in the gums and surrounding tissues, in the classification of periodontal diseases, in the detection of plaque with intraoral photographs and fluorescent imaging, in the diagnosis of oral microbiota, and in the classification of halitosis. The use of AI techniques in combination with different types of data for diagnosing periodontal diseases has been extensively investigated in the literature.^2^ An algorithm that detects periodontal bone loss from PRs with the trained CNN model was developed in a study. This model was compared with clinical data, and AI was reported to detect periodontal bone loss successfully. However, a negative situation that emerged in this study is the poor detection of periodontal bone loss in the third molar. The reason for this is that the sample image data of the third molar is low when forming the AI model.^46^ Lin et al^47^ reported in their study that automatic alveolar bone loss areas could be detected in periapical films; in another study, they developed a model that could measure the degree of alveolar bone loss.^48^ Lee et al^49^ developed a DL-based CNN model for the automatic detection of periodontally weakened premolar and molar teeth. In this study, they stated that there was a higher diagnostic accuracy in the premolar teeth, and they thought this might be because the molar teeth have 2 or 3 roots and anatomical variations.

If the bacteria that attach to the teeth and surrounding tissues colonize in an organized layer and form a biofilm, this is called a plaque. Dental plaque can occur on tooth surfaces for many reasons. The oral microbiota that forms the plaque may vary from person to person or even in different oral regions of the same individual.^50^ Artificial intelligence-based oral microbiota analysis related to periodontal diseases was performed, and 4 different ML methods (RF, DL, SVM, and logistic regression) were used to evaluate the patient’s periodontal health. The RF algorithm had the best performance.^51^ Mason et al^52^ conducted a study that could predict ethnicity from microbial colonization found in dental plaque and saliva samples and used the RF algorithm for this study. This study can explain the cause of different microflora in the oral region of individuals of different ethnicity.

Fluorescent biomarkers are used in the diagnosis of oral cancer and retinal diseases, as well as in the detection of periodontal disease and dental plaque. The biomarker has a high degree of accuracy despite certain disadvantages.^53^ The AI model, trained by fluorescent biomarkers and experts, has been shown to detect the presence and location of dental plaque with high sensitivity in intraoral photographs.^54^ Thus, they have developed an AI model that can help plaque control with intraoral images and provide early periodontal disease detection without various plaque imaging methods or experts. Another study produced an automatic AI model that can distinguish healthy and inflamed gums from fluorescent images obtained using intraoral cameras.^55^ With the developed model, intraoral images can detect gingivitis, preventing the formation of advanced periodontal diseases.

Halitosis is a bad smell that comes from the inside or outside the mouth. In daily life, dentists are the first people the patients affected by bad breath apply to. Although there is no universal classification in terminology for halitosis, it is an issue that has been studied in different ways in the literature. In general, halitosis is classified as primary and secondary. Primary halitosis is caused by lung respiration, and the mouth and upper respiratory tract cause secondary halitosis.^56^

Nakano et al^57^ used ANN, SVM, and decision trees to classify bad odor in the mouth from oral microbiota and methyl mercaptan levels in saliva and classified odor with relatively low accuracy. In another study conducted by the same team, it was observed that the accuracy and sensitivity in classifying halitosis using DL increased compared to the previous study.^58^

## Oral Diagnosis and Maxillofacial Radiology and Artificial Intelligence

Due to the frequent use of pre-treatment imaging systems in dentistry and the high radiographic data, many suitable areas have formed for AI studies. Correct examination of radiograms depends on the ability of dentists to interpret images. Therefore, many AI studies can help the dentist in accurate and rapid diagnosis in radiological examinations. However, since the same standardization cannot always be achieved in radiological images and due to the complexities and errors in the images, AI cannot fully replace human intelligence in the interpretation of radiograms.

Egger et al.^59^ on CT images, and Wang et al.^60^ on CBCT images, developed a fully automatic AI model to segment the mandible in the craniomaxillofacial region. Some studies conducted to segment the mandibular canal have focused on CT images and^61^ on CBCT images.^62^ In another study, the U-net CNN model was used to automatically detect the relationship of the third molar teeth with the inferior mandibular canal using the PR images’ data and segmenting it.^63^

Artificial intelligence models have been developed that automatically detect and/or classify teeth using periapical and bitewing radiographs.^64,65^ In the study with bitewing radiographs in the data set, parameters such as the shape of the pulp, width–height ratios, and crown size were evaluated in SVM and sequence alignment algorithms.^66^ The missing aspect of this study is that the incisors and canine teeth were not included. In PRs, AI studies can also segment^67^ and number teeth^68^ other than the third molar. In many similar studies, algorithms that can perform tooth segmentation and/or numbering in PR have been developed using different methods.^69,70^ In a study, the CNN model, which can be classified as molar, premolar, canine, and incisor after defining the oral cavity in PRs, was developed, and its accuracy was reported to be more than 90.0%.^71^ Accuracy was found to be partially low for the premolars, and they stated this was due to the similarity with the adjacent canine teeth.

There are also studies in which tooth segmentation was performed on CBCT images. For example, Miki et al^72^ developed a deep CNN model with 91.0% accuracy, which can classify tooth groups other than the third molar in CBCT images. The data used in the study consist of axial images with only 1 tooth. In another study conducted by the same researchers, unlike their previous studies, a model that can automatically classify teeth from axial images without image cutting was developed.^73^ In their study, Hosntalab et al^74^ developed an algorithm that can automatically classify and number according to the contour of the teeth in CT images without using anatomical features such as the order or estimated location of the tooth. Thus, more successful tooth segmentations can be performed for missing teeth, early tooth extraction, and tooth anomalies.

In 1992, Mol and Van Der Stelt^75^ developed a computer-aided image analysis system that defines the periapical region in dental radiographs, determines the presence of periapical lesions, and estimates the size of the structure in the presence of lesions. In their study, Orhan et al^76^ used the CNN model to measure the periapical lesion’s detection, localization, and volume in CBCT images. The periapical lesion detection rate of the AI model used in this study was 92.8%.

Temporomandibular joint (TMJ) diseases are disorders in joint functions resulting from intra-articular or extra-articular pathologies.^77^ Using the characteristic clinical signs and symptoms of TMJ diseases, AI was used to divide TMJ internal irregularities into 2 subgroups (reduction anterior disc displacement and non-reduction anterior disc displacement) and to predict healthy joints.^78^ In a study conducted by Nam et al.^79^ they used an AI-assisted system to distinguish the diseases imitating TMJ dysfunctions from real TMJ diseases and stated that this system has the potential to help clinicians.

The maxillary sinus is an anatomical cavity filled with air symmetrically located on both sides of the nasal cavity in the upper jawbone. Knowing about the maxillary sinuses normal volumes, anatomy, and variations is extremely important for dental treatments in the maxilla’s posterior region. Clinical examination and traditional radiography methods diagnose and evaluate pathologies in maxillary sinuses.^80^ In their study, Kuwana et al^81^ developed an AI model that detects maxillary sinus localization and lesions in the relevant sinuses in PRs. In their study, Murata et al^82^ used the CNN model to detect maxillary sinusitis in PRs. Artificial intelligence accuracy, sensitivity, and specificity were 87.5%, 86.7%, and 88.3%, respectively. They compared these values with radiologists and general dental practitioners and reported that AI performed higher than general dental practitioners. In another study, a model that can help diagnose sinusitis using computer-aided detection in PRs was developed.^83^ The negative aspect of this study is that it can detect the presence of unilateral sinusitis. Using these models, useful information can be given to dentists to diagnose inflammatory diseases in the sinus. In addition, important clues can be presented to clinicians in the differential diagnosis of dental pain and maxillary sinusitis.

Osteoporosis or osteoporosis means loss of robustness and tissue organization due to the appearance of several changes in the structure of bone tissues due to various factors. This disease, which affects many people, especially women, and causes pathological fractures, has led researchers to diagnose the disease with dental radiographs because other diagnostic methods are expensive.^84^ Many AI studies have been conducted to help diagnose osteoporosis from dental PRs.^85,86^ Kavitha et al^85^ have developed an automatic detection system based on the geometric properties of the cortical and trabecular bone of the mandible in PRs to detect female patients with low bone mineral density or osteoporosis. In their study, Hwang et al^86^ selected several regions of interest (ROI) for the diagnosis of osteoporosis in PRs and used strut analysis, fractal size, and gray-level co-occurrence matrices and evaluate their performance. In the classification model developed using the decision tree and SVM, they reported that the most suitable region for disease detection from the selected ROI regions (center of the condyle head, center of the ramus, area between the first and second molar apices, endosteal margin region) was the endosteal margin region and the best performance was demonstrated by strut analysis. The proliferation and presence of dental panoramic devices in many centers can help patients diagnose osteoporosis with the help of AI models and direct them to relevant experts.

In radiological examinations, a good radiographic image is as important as the knowledge and experience of the physician for the correct interpretation of the image. Various AI models were used for this purpose. In PRs, the CNN model, which can predict the positioning error of the dental arch in the anterior and posterior direction, was developed.^87^ Thus, it was aimed to reduce the number of blurred images that may occur due to the arc position. There are also studies conducted to reduce artifacts in CBCT images.^88^ Hatvani et al^89^ studied 2 different CNN models to increase the resolution in CBCT image slices and stated that they obtained remarkable results. Hu et al^88^ used generative adversarial networks (GAN), CNN, and mean Wasserstein distance (m-WGAN) models to correct the artifacts formed in CBCT images and to form a better image for diagnosis. They reported that m-WGAN performed best in artifact reduction and increasing the detail in the images.

Tooth age is one of the methods that can be estimated based on the development stages of the teeth observed in radiographs. Evaluating these stages with radiographs is more advantageous than the clinical evaluation of teeth affected by local and systemic factors. Panoramic radiographs were used to determine the tooth age according to the degree of calcification observed in radiographic examinations of permanent teeth.^90^ Age estimation can also be made from dental x-rays using the AI method.^91^ Velemínská et al.^91^ in a study in which they estimated age from PRs, used PR images of individuals between the ages of 3 and 17 in the Czech population and estimated age according to the developmental stages of mandibular teeth.

Dental implant treatment is a very effective method for rehabilitating tooth loss, and its popularity has increased in recent years. Today, there are many implant models and types. Implants consist of fixtures, abutments, and superstructures that can vary according to the model, shape, and necessary tools.^92^ The problems in recording the implant brands and models applied to prevent the continuation of the treatment in terms of prosthetics. Therefore, it is important to identify the implant brand correctly. Many AI studies have been carried out for implant brand detection. Studies have tried to determine the implant model by using periapical^93^ or PRs.^94^ Liu et al^95^ developed a model that can predict the failure of dental implants.

## Orthodontics and Artificial Intelligence

Accurate diagnosis is very important in orthodontic applications aiming to correct the irregularities in the teeth, the relationships of the jaws with each other, malocclusions, and the positions of the jaw bones on the facial skeleton. Malocclusions are common, and this is a serious public health problem in developed countries. Therefore, the causes and etiologies of malocclusions should be investigated.^96^

Many AI studies have been conducted in the field of orthodontics. Artificial intelligence studies include various stages of orthodontic studies such as Landmark detection, skeletal classification, treatment planning, and help dentists. Thanks to these studies, orthodontists can evaluate patients more quickly and accurately before treatment.

Cephalometric analysis is an important part of orthodontic diagnosis and treatment planning. It provides important information about the relationships between dental-maxillofacial structures that cannot be easily obtained by physical examination.^97^ Artificial intelligence was used for orthodontic analysis and landmark detection, and the landmarks, angles, lengths, and orthodontic ratios determined by orthodontic specialists and the developed AI model were compared in cephalometric films.^98^ It was stated that there was no significant difference between AI and specialists. In a study comparing the landmarks used in lateral cephalometric analysis with the DL methods, you-only-view-forget version 3 (YOLOv3), and single-shot multibox detector (SSD) AI methods, the accuracy of YOLOv3 was found to be higher than SSD.^99^ Some studies make a skeletal classification in lateral cephalograms using the SVM method.^100^ Using approximately 5900 lateral cephalometric radiographs, an AI model that automatically determines skeletal classification and eliminates the landmark detection process was developed.^101^ In this modeling, it was reported that AI showed sensitivity and accuracy above 90.0% in vertical (hyperdivergent and hypodivergent) and sagittal (class I, class II, and class III) skeletal classification. It was also found that the accuracy rate was higher in vertical classification compared to sagittal classification. An algorithm has been developed to automatically detect 20 cephalometric points on the images obtained with CBCT.^102^ In another study, it was revealed that the automatic detection of cephalometric landmarks in CBCT images and the determined measurements with the developed algorithm had great similarity with manual determinations and measurements.^103^

The degree of skeletal development reflects a person’s degree of physiological maturation. It is known that bone age is as important as chronological age in evaluating the physical development of an adolescent.^104^ Since wrist radiographs provide common findings on a single image, they are an ideal data set for bone age determination with AI. Many studies determine the automatic bone age by looking at the wrist images.^105^ Lee et al^106^ determined bone age with high accuracy in the CNN model they developed and stated that this system is a much faster and more efficient decision support system than traditional methods. On the other hand, Yune et al^107^ reported that they distinguished gender by the left hand-wrist radiographs of approximately 10 600 patients between the ages of 5 and 70 with the help of the deep CNN model. Thus, AI can define a situation that cannot be distinguished by human intelligence and the eye with its algorithm.

Developmental dental anomalies are common in orthodontic patients. Anomalies in the number, shape, and position of teeth may cause disorders in maxillary and mandibular arch length and occlusion, making orthodontic treatment planning difficult.^108^ Orthodontic treatments may include closing the cavities in the dental arch and opening the cavity for prosthetic replacement or implant in the arch.^109^ For this purpose, sometimes, a decision can be made for orthodontic treatment. Artificial intelligence models have been developed that can help make this decision. In the AI model, which was formed to decide whether the extraction treatment would be effective in orthodontic patients between the ages of 11 and 15, the accuracy rate was reported to be around 80%.^110^

Orthognathic surgery is the most effective treatment to eliminate skeletal problems after puberty. In their study, Patcas et al^111^ developed an AI model to define the effect of orthognathic treatment on facial attraction and visible age. They formed the AI model they used by using the database of a dating site with more than 13 000 face images with more than 17 million ratings. They concluded that the patients looked 1 year younger, and their attractiveness increased by 74.7%.

## Pediatrics Dentistry and Artificial Intelligence

It is important that the diagnosis is accurate to determine the most appropriate treatment procedure in dentistry. Especially in pediatric dentistry, faster and more effective diagnoses enable patients to cooperate better and increase the success rate. Dentists commonly use periapical and PRs for diagnosis.^112^

Recently, AI sub-based systems have been developed to prevent dentists from overlooking dental problems and increase the accuracy of radiological diagnoses. In their study, Kılıç et al^113^ evaluated the success of the DL method in the automatic detection and numbering of milk teeth and used 421 PRs of pediatric patients between the ages of 5 and 7 for this purpose.

In their study, Kaya et al^114^ experienced the deep CNN algorithm YOLOv4 to detect permanent dental germs in 4518 PR of pediatric patients between the ages of 5 and 12 and thus aimed to reach the correct diagnosis by reducing workflow and human-induced errors.

Calıskan et al^115^ used CNN algorithms to detect and classify impacted milk molars in PR images of 74 pediatric patients aged 5 to 12 years and reported that the system had high accuracy. Ahn et al^116^ used 4 different DL methods using a total of 1100 images with and without 550 mesiodens to detect mesiodens in PRs of milk and mixed dentition period. They reported that ResNet-101 and Inception-ResNet-V2 models performed better. In this study, it is thought that AI-supported PRs will help clinicians to detect mesiodens, and early diagnosis of these teeth will reduce future dental complications. Ha et al^117^ aimed to develop an AI model that detects mesiodens in PRs of different dentition periods. For this purpose, 612 PRs were used, and a CNN model based on YOLOv3 was developed to detect mesiodens. The proposed model has performed well and has the potential for clinical use to detect mesiodens in PRs of all dentition periods.

## Prosthetic Dentistry and Artificial Intelligence

With prosthetic dental treatment, loss of substance in natural teeth, missing teeth, and oral and maxillofacial tissue defects are restored with artificial materials. It aims to correct and maintain oral functions, phonation, aesthetics, and patient health. The preparation stages of prosthetic restorations are procedures that can be carried out by conventional or digital methods and require high precision.^118^ Computer-aided design/computer-aided manufacturing (CAD/CAM) refers to a production technique in which computer skills are used to design and produce prosthetic parts. In recent years, CAD/CAM has been widely used in producing fixed prosthetic restorations. Smart software tools are needed to optimize the digital digits of CAD/CAM systems. With the development of digital systems and the widespread use of AI in dentistry, AI in prosthetic treatments has also increased.^119,120^

The CAD/CAM systems greatly affect the planning and construction of prostheses in prosthetic treatments. However, it may not always be easy to reach this technology. In all 3 studies, the similarity of occlusal morphology between the reconstructions made by the AI model integrated into the dental CAD software program for 1 restoration and the original dental morphology or hand-made reconstructions completed by the dental laboratory technician was analyzed. The results showed that a successful AI model was formed.^121^

Measuring with measuring spoons and obtaining a model is a method that is frequently done and easily accessible within the routine. In AI studies, tooth classification and segmentation were planned through 3D models.^122^ Thus, easily obtained in clinical applications, the plaster model can be included in an automated process chain. Accordingly, Raith et al^122^ detected teeth’ segmentation and the localization of tubercle peaks in plaster models digitized by 3D scanning using AI.

Although the popularity of implant applications has increased in eliminating tooth deficiencies, removable prostheses are still used as the primary treatment. One of the reasons for this is that these prostheses are cheaper. It is very important to design removable prostheses appropriately. Depending on the design, patients may have discomfort, esthetic problems, and, more importantly, problems such as not using the prosthesis. For this purpose, Chen et al^123^ developed a clinical decision support model to design removable prostheses. In their study, in which they stated that they developed an ontological paradigm, they developed an algorithm to calculate the similarity value between the patients provided as input and ontology cases.

While the current use of AI is increasing, it consumes almost all the data in dentistry. In this review, current AI applications in dentistry are reviewed and prominent researches are mentioned. In these studies examined,

- Artificial neural network model has been used most in AI studies in dentistry.
- Artificial intelligence studies in dental practice have generally focused on the evaluation of diagnosis and diagnostic methods based on digital radiological data. This is because the number of data to be used to develop AIs that accelerate diagnosis and help in appropriate treatment planning is more readily available. It is also the ability of AI to detect changes at the pixel level that the human eye cannot distinguish or notice.
- Differential diagnosis of various diseases and lesions in the maxillofacial region is difficult for physicians. More studies have been carried out in the field of oral pathology and oral diagnosis in order to solve this complex situation and reduce the need for specialist physicians, generally using digital radiological images and intraoral photographs.
- Since specialists and special programs are needed in the analysis required for the detection of skeletal and dental malocclusions and making the treatment planning decision in orthodontic treatments, AI studies in the related field have been about meeting these requirements and making faster analysis.
- In pediatric dentistry, AI studies have been done less than in other fields, and they are generally on the detection of primary and permanent tooth segmentation and dental anomalies in digital images.

In conclusion, technology has a great place in our lives and makes our daily lives easier. Artificial intelligence techniques and applications are still in use, constantly developing and giving hope for the future.

Artificial intelligence cannot replace the human role in dentistry, but it helps use appropriate treatments to diagnose and diagnose diseases. The variety of models, applications, and data used in AI studies, as well as the difference in the knowledge and experience of the people working on training the models, prevents AI from reaching a certain standard and causes statistical differences. Providing certain standardization, using larger data sets, and conducting multidisciplinary studies by field experts will increase the use and popularity of AI in dentistry.
